# Supplementary material for: Inference of fitness landscapes with heterogeneous patterns of epistasis across sites
Source: bioRxiv. 2026 Jun 28:2026.06.25.734428. Preprint. [Version 1] doi: 10.64898/2026.06.25.734428 (PMC13320847; doi:10.64898/2026.06.25.734428)
Supplement: Supplement 1 [file NIHPP2026.06.25.734428v1-supplement-1.pdf]

bioRxiv preprint doi: https://doi.org/10.64898/2026.06.25.734428; this version posted June 28, 2026. The copyright holder for this preprint (which was not certified by peer review) is the author/funder, who has granted bioRxiv a license to display the preprint in perpetuity. It is made available under a CC-BY 4.0 International license.

Supplementary Information

Epistasis on fitness landscapes

Let f be an α^ℓ-dimensional vector encoding the fitness associated with each genotype in the space of possible haploid sequences with α alleles and ℓ sites $S = \{1, 2, \ldots, \ell\}$. In this section, we review two common ways to quantify epistasis in a fitness landscape: one based on local epistatic coefficients (Zhou and McCandlish 2020; Chen *et al.* 2021) and a second one based on the variance explained by genetic interactions of different orders (Stadler and Happel 1999; Zhou *et al.* 2022) or across subsets of sites (Martí-Gómez *et al.* 2026b).

Variance components Any fitness landscape f can be decomposed into orthogonal components f_k corresponding to genetic interactions of order k

$$f = \sum_{k=0}^{\ell} f_k. \tag{S1}$$

These components can be obtained by projecting the f into the kth-subspace using the orthogonal projection matrix P_k given by the Krawtchouk polynomials (Stadler and Happel 1999; Zhou *et al.* 2022):

$$P_k(x, x') = \alpha^{-\ell} \sum_{q=0}^{k} (-1)^q (\alpha - 1)^{k-q} \binom{d(x, x')}{q} \binom{\ell - d(x, x')}{k - q}. \tag{S2}$$

Moreover, each k-th order subspace can be further decomposed into orthogonal components corresponding to the contributions of interactions between specific subsets of sites $U \subseteq S$ of size k:

$$f = \sum_{U \in \mathcal{P}(\mathcal{S})} f_U, \tag{S3}$$

where $\mathcal{P}(\mathcal{S})$ is the set of all subsets of S (i.e. the power set of S) and $f_U = P_U f$, where P_U is an orthogonal projection matrix onto the corresponding subspace. Each such P_U can be expressed as a Kronecker product of a series of site-specific projection matrices onto the constant subspace, defined by $P_{\text{con}} = \frac{1}{\alpha} 1 1^T$, and the orthogonal or additive subspace, given by $P_{\text{add}} = I - P_{\text{con}}$. In particular

$$P_U = \bigotimes_{i=1}^{\ell} P_{i \in U}, \tag{S4}$$

where $P_{i \in U} = P_{\text{add}}$ when site $i \in U$ and $P_{i \in U} = P_{\text{con}}$ otherwise (Martí-Gómez *et al.* 2026b):

$$P_U(x, x') = \alpha^{-\ell} \prod_{i \in U : x_i = x'_i} (\alpha - 1) \prod_{i \in U : x_i \neq x'_i} (-1). \tag{S5}$$

Because U-components are orthogonal to each other, the total variance in a fitness landscape can be expressed as a sum of variances explained by genetic interactions of order k (i.e. summing over all U with $|U| = k$) or by interactions between the sites U, respectively (Martí-Gómez *et al.* 2026b):

$$\text{Var}[f] = \sum_{k=1}^{\ell} \text{Var}[f_k] = \sum_{U \in \mathcal{P}(S)} \text{Var}[f_U]. \tag{S6}$$

Local epistatic coefficients The classical way to quantify epistasis is through the definition of the epistatic coefficient ϵ for a pair of mutations at different loci $A \to a$ and $B \to b$, which quantifies the difference in the effect of mutation $A \to a$ in the presence of allele B compared to that in the presence of allele b at the other locus:

$$\epsilon = (f_{AB} - f_{aB}) - (f_{Ab} - f_{ab}). \tag{S7}$$

This epistatic coefficient is defined locally, as it depends only on four specific genotypes sharing the same genetic background. Still, we can compute the average magnitude of local epistatic interactions $\overline{\epsilon^2}$ in a given fitness landscape by averaging the its squared values across every possible pair of mutations at every possible genetic background:

$$\overline{\epsilon^2} = \frac{1}{s} f^T \Delta^{(2)} f, \tag{S8}$$

where $s = \binom{\ell}{2}\binom{\alpha}{2}^2 \alpha^{\ell-2}$ is the number of epistatic coefficients and $\Delta^{(2)}$ is a positive semi-definite sparse matrix (Zhou and McCandlish 2020; Chen *et al.* 2021). These results can be generalized to quantify local epistatic coefficients of any order P using

$$\Delta^{(P)}(x, x') = \begin{cases} 0 & \text{if } d(x, x') > P \\ (-1)^{d(x,x')} (\alpha - 1)^{P - d(x,x')} \binom{\ell - P}{P - d(x,x')} & \text{if } d(x, x') \leq P, \end{cases} \tag{S9}$$

where $d(x, x')$ represents the Hamming distance i.e. number of single point mutations, separating sequences x and x', such that the sum of the squared P-th order local epistatic coefficients is given by $f^T \Delta^{(2)} f$ and the mean sqaured P-th order local epistatic coefficient

bioRxiv preprint doi: https://doi.org/10.64898/2026.06.25.734428; this version posted June 28, 2026. The copyright holder for this preprint (which was not certified by peer review) is the author/funder, who has granted bioRxiv a license to display the preprint in perpetuity. It is made available under a CC-BY 4.0 International license.

is obtained by dividing by the number of such coefficients $\binom{\ell}{P}\binom{\alpha}{2}^P\alpha^{\ell-P}$ (Chen *et al.* 2021). Interestingly, $\Delta^{(P)}$ can also be expressed as a weighted sum of the projection operators into the k-th order subspaces given by P_k:

$$\Delta^{(P)} = \sum_{k=0}^{\ell} \lambda_k P_k = \sum_{k=P}^{\ell} \lambda_k P_k, \quad \text{(S10)}$$

where $\lambda_k = \alpha^P \binom{k}{P}$ (Chen *et al.* 2021) correspond to the eigenvalues of the $\Delta^{(P)}$ operator. Note that the second equality arises because $\binom{k}{P} = 0$ for $k < P$, corresponding to the fact that all local P-th order epistatic coefficients are zero for any function f with maximal order less than P (so that all such functions are in the null space of $\Delta^{(P)}$) and that moreover alteration of any component of order less than P for an arbitrary f leaves all P-th order local epistatic coefficients unchanged.

Epistatic coefficients among subsets of sites

In this section, we describe how the $\Delta^{(P)}$ operator that extracts the sum of squared epistatic interactions in a fitness landscape can be decomposed into the sum of simpler operators that we call $\Delta^{(U)}$, corresponding to local epistatic interactions only among a subset of sites $U \subseteq S$. These results generalize the main text results focused on local epistatic interactions between pairs of sites i and j i.e. $U = \{i, j\}$.

Let E_U be a $s_U \times \alpha^\ell$ matrix such that the entries in the $E_U f$ vector encode all the local $|U|$-th order epistatic coefficient for a subset of positions U. s_U is the number of different epistatic coefficients corresponding to local $|U|$-th order mutant epistatic coefficients within sites U, and is given by the product of the number of genetic backgrounds at sites not in U $\alpha^{\ell-|U|}$ and the number of possible combinations of mutations across the sites U $\binom{\alpha}{2}^{|U|}$:

$$s_U = \alpha^{\ell-|U|}\binom{\alpha}{2}^{|U|} = \alpha^{\ell-|U|}\left(\frac{\alpha(\alpha-1)}{2}\right)^{|U|} = \frac{\alpha^\ell}{2^{|U|}}(\alpha-1)^{|U|}. \quad \text{(S11)}$$

Thus,

$$\overline{\epsilon_U^2} = \frac{1}{s_U}(E_U f)^T E_U f = \frac{1}{s_U} f^T \Delta^{(U)} f, \quad \text{(S12)}$$

where the entries of $\Delta^{(U)}$ for a pair of sequences x and x' can be obtained by summing over all possible local $|U|$-mutant epistatic coefficients between sites in U

$$\Delta^{(U)}(x, x') = \sum_{m=1}^{s_U} E_U(m, x) E_U(m, x'). \quad \text{(S13)}$$

$E_U(m, x') = 0$ if sequence x' is not involved in epistatic coefficient m, and takes values -1 or 1 otherwise. Thus, we only need to sum overlocal epistatic coefficients involving both x and x'. If $x_i \neq x'_i$ for any position $i \notin U$, then sequences x and x' cannot be involved in any epistatic coefficient and thus $\Delta^{(U)}(x, x') = 0$. Otherwise, $E_U(m, x)E_U(m, x') = (-1)^{d(x,x')}$ depending on the Hamming distance between them $d(x, x')$. Moreover, the number of local $|U|$-mutant epistatic coefficients that involve both sx and x' is given by $(\alpha-1)^{|U|-d(x,x')}$. Thus

$$\Delta^{(U)}(x, x') = \begin{cases} (-1)^{d(x,x')}(\alpha-1)^{|U|-d(x,x')} & \text{if } x_i = x'_i\ \forall i \notin U \\ 0 & \text{otherwise.} \end{cases} \quad \text{(S14)}$$

We can verify that we can recover the $\Delta^{(P)}$ operator by summing $\Delta^{(U)}$ over all possible subsets of sites U of size P. If $d(x, x') > P$, there is no single set of sites U for which the context at sequences x and x' can match and thus $\sum_{U:|U|=P} \Delta^{(U)}(x, x') = 0$. For $d(x, x') \leq P$, the entries of $\Delta^{(U)}(x, x')$ are the same but they are summed over multiple U. As we only sum over U's such that x and x' share the context, the number of times we are summing them corresponds to the number of ways we can choose $P - d(x, x')$ sites that match out of the $\ell - P$ sites in the shared context between them. Thus,

$$\sum_{U:|U|=P} \Delta^{(U)}(x, x') = \begin{cases} (-1)^{d(x,x')}(\alpha-1)^{P-d(x,x')}\binom{\ell-P}{P-d(x,x')} & \text{if } d(x, x') \leq P \\ 0 & \text{if } d(x, x') > P, \end{cases} \quad \text{(S15)}$$

which exactly matches the $\Delta^{(P)}$ operator (Zhou and McCandlish 2020; Chen *et al.* 2021).

Relationship between local epistatic coefficients and variance components

In this section, we describe some properties of the new $\Delta^{(U)}$ operator and the relationships with the projection operators into the subspaces corresponding to interactions of different orders and subsets of sites U.

One useful property of the $\Delta^{(U)}$ operator is that it can be expressed as a Kronecker product of site-specific matrices

$$\Delta^{(U)} = \bigotimes_{i=1}^{\ell} \Delta_{i\in U} = \bigotimes_{i=1}^{\ell} \begin{cases} \alpha P_{\text{add}} & \text{if } i \in U \\ I & \text{if } i \notin U, \end{cases} \quad \text{(S16)}$$

bioRxiv preprint doi: https://doi.org/10.64898/2026.06.25.734428; this version posted June 28, 2026. The copyright holder for this preprint (which was not certified by peer review) is the author/funder, who has granted bioRxiv a license to display the preprint in perpetuity. It is made available under a CC-BY 4.0 International license.

with entry-wise formula given by:

$$\Delta^{(U)}(x,x') = \prod_{\substack{i\in U\\ x_i=x'_i}} (\alpha-1) \prod_{\substack{i\in U\\ x_i\neq x'_i}} (-1) \prod_{\substack{i\notin U\\ x_i=x'_i}} (1) \prod_{\substack{i\notin U\\ x_i\neq x'_i}} 0. \tag{S17}$$

Thus, we can use the mixed-product property to show that the columns of $P_{U'}$ are eigenvectors of $\Delta^{(U)}$ with eigenvalue $\alpha^{|U|}$ if $U \subseteq U'$ or are in the null space of $\Delta^{(U)}$ whenever $U \not\subseteq U'$.

$$\Delta^{(U)}P_{U'} = \bigotimes_{i=1}^{\ell} \Delta_{i\in U} \bigotimes_{i=1}^{\ell} P_{i\in U'} = \bigotimes_{i=1}^{\ell} \Delta_{i\in U} P_{i\in U'}, \tag{S18}$$

where

$$\Delta_{i\in U}P_{i\in U'} = \begin{cases} P_{\text{con}} & \text{if } i\notin U \land i \notin U' \\ P_{\text{add}} & \text{if } i\notin U \land i \in U' \\ 0 & \text{if } i\in U \land i \notin U' \\ \alpha P_{\text{add}} & \text{if } i\in U \land i \in U'. \end{cases} \tag{S19}$$

Therefore

$$\Delta^{(U)}P_{U'} = \begin{cases} \alpha^{|U|}P_{U'} & \text{if } U\subseteq U' \\ 0 & \text{if } U \not\subseteq U'. \end{cases} \tag{S20}$$

Because the projection operators $P_{U'}$ are orthogonal to each other, we can express $\Delta^{(U)}$ as a linear combination of $P_{U'}$ weighted by their eigenvalues (either 0 or $\alpha^{|U|}$):

$$\Delta^{(U)} = \alpha^{|U|} \sum_{U':U\subseteq U'} P_{U'}, \tag{S21}$$

so that, using the fact that sums of projection matrices into orthogonal subspaces are themselves projection matrices, we see that $\Delta^{(U)}$ is itself just a $|U|$-dependent constant times a specific projection matrix. Moreover, this relation between $\Delta^{(U)}$ and the $P_{U'}$ with $U \subseteq U'$ can be used to derive the relationship between the $\Delta^{(P)}$ operator and the projection operator into the kth order subspace P_k:

$$\Delta^{(P)} = \sum_{U:|U|=P} \Delta^{(U)} = \sum_{U:|U|=P} \alpha^{|U|} \sum_{U':U\subseteq U'} P_{U'} = \alpha^P \sum_{U:|U|=P}\sum_{U':U\subseteq U'} P_{U'}. \tag{S22}$$

It is now easy to see that the number of times we are summing each $P_{U'}$ depends on the size of U'. In particular, it corresponds to the number of $U : |U| = P$ subsets in U', which can be calculated as the number of ways of choosing $|U| = P$ sites out of $|U'|$. Then, we can use the fact that $P_k = \sum_{U:|U|=k} P_U$ (Martí-Gómez *et al.* 2026b) to recover the eigendecomposition of the $\Delta^{(P)}$ operator (Chen *et al.* 2021):

$$\Delta^{(P)} = \alpha^P \sum_{U':|U'|\geq P} \binom{|U'|}{P} P_{U'} = \sum_{k\geq P} \alpha^P \binom{k}{P} \sum_{U':|U'|=k} P_{U'} = \sum_{k\geq P} \alpha^P \binom{k}{P} P_k. \tag{S23}$$

Relationship between local $|U|$-way epistatic coefficients and the epistatic variance explained by the subset U

A useful low dimensional summary statistic to describe the patterns of genetic interactions across subsets of sites in a fitness landscape is the epistatic variance explained by a set of sites U (Crawford *et al.* 2017; Reddy and Desai 2021; Martí-Gómez *et al.* 2026b). We define this variance $\text{Var}^{(U)}[f]$ to include not only the variance explained by $|U|$-way interactions between mutations at the sites U, but also all other interactions of order higher than $|U|$ where all sites in U are involved, so that $\text{Var}^{(U)}[f]$ provides an overall measure of the amount of $|U|$-way and higher epistasis that the subset of sites U is involved in. This quantity can be computed by projecting the fitness landscape f onto the 2^ℓ subspaces defined by every possible U' given by $f_{U'} = P_{U'}f$, summing over the $f_{U'}$'s of interest and computing the inner product.

$$\text{Var}^{(U)}[f] = \left(\sum_{U':U\subseteq U'} f_{U'}\right)^T \left(\sum_{U':U\subseteq U'} f_{U'}\right) = \left(\sum_{U':U\subseteq U'} P_{U'}f\right)^T \left(\sum_{U':U\subseteq U'} P_{U'}f\right) = f^T\left(\sum_{U':U\subseteq U'} P_{U'}\right) f, \tag{S24}$$

where

$$\sum_{U':U\subseteq U'} P_{U'} = \sum_{U':U\subseteq U'} \bigotimes_{i=1}^{\ell} P_{i\in U'}. \tag{S25}$$

As the Kronecker product is commutative up to row and column permutations given by Q_1 and Q_2, we can write the factors in order depending on whether the sites are in U or not:

$$\begin{aligned}\sum_{U':U\subseteq U'} P_{U'} &= Q_1\left(\sum_{U':U\subseteq U'} \bigotimes_{i\in U} P_{i\in U'} \bigotimes_{i\notin U} P_{i\in U'}\right) Q_2 = Q_1\left(\bigotimes_{i\in U} P_{\text{add}} \sum_{U':U\subseteq U'} \bigotimes_{i\notin U} P_{i\in U'}\right) Q_2 \\ &= Q_1\left(\bigotimes_{i\in U} P_{\text{add}} \bigotimes_{i\notin U} I\right) Q_2 = \bigotimes_{i=1}^{\ell} \begin{cases} P_{\text{add}} & \text{if } i\in U \\ I & \text{if } i\notin U \end{cases} = \alpha^{-|U|}\Delta^{(U)}. \end{aligned} \tag{S26}$$

bioRxiv preprint doi: https://doi.org/10.64898/2026.06.25.734428; this version posted June 28, 2026. The copyright holder for this preprint (which was not certified by peer review) is the author/funder, who has granted bioRxiv a license to display the preprint in perpetuity. It is made available under a CC-BY 4.0 International license.

Thus, this shows that the sum of squared epistatic coefficients among a subset of sites U given by $f^T\Delta^{(U)}f$ is equal to the epistatic variance explained by those sites $\mathrm{Var}^{(U)}[f]$ multiplied by a factor of $\alpha^{|U|}$. Noting that $f^T\Delta^{(U)}f = \overline{\epsilon^2_U}s_U$ and simplifying $s_U/\alpha^{|U|}$ yields Equation 2 in the main text. Similarly, the sum of squared local P-epistatic coefficients across the complete fitness landscape given by $f^T\Delta^{(P)}f$ can also be interpreted as the sum the epistatic variances of all possible combinations of P sites multiplied by a factor of α^P.

Relationship between mean and variance of local $|U|$-way epistatic coefficients and the variance components

In the previous sections, we have shown the relationships between the average squared epistatic coefficients between mutations at sites U and the total variance explained by interactions of order $|U|$ or larger involving all sites in U. In this section, we describe how these quantities relate to the mean and variance of the distribution of $|U|$-way epistatic coefficients for a set of mutations within sites U. To do so, we first decompose the epistatic coefficients between mutations at sites U into the epistatic coefficients for each possible combination of mutations at sites U. Using this more granular statistic, we derive the mean of the epistatic coefficients between a given set of mutations, and use its square, together with the average squared epistatic coefficient to compute the variance. Finally, we take the average of these variances over all possible combinations of mutations at sites U and derive its relationship to the variance components defined over the subsets of sites U.

Let $C = \{\{c_{11}, c_{12}\}, \dots, \{c_{k1}, c_{k2}\}\}$ be the set of k pairs of characters at a set of k positions U and ϵ_{UC} be the $\alpha^{\ell-k}$-dimensional vector of k-way epistatic coefficients between the mutations specified by C across every possible genetic background. Let E_{UC} be an $\alpha^{\ell-k} \times \alpha^{\ell}$ such that $\epsilon_{UC} = E_{UC}f$ and note that this matrix that can be expressed as a Kronecker product of site specific factors:

$$E_{UC} = \bigotimes_i^{\ell} \begin{cases} E_i^{c_{i1},c_{i2}} & \text{if } i \in U \\ I & \text{if } i \notin U, \end{cases} \tag{S27}$$

where $E_i^{c_1,c_2}$ is a row vector with entries given by

$$E_i^{c_1,c_2}(x) = \begin{cases} 1 & \text{if } x_i = c_1 \\ -1 & \text{if } x_i = c_2 \\ 0 & \text{otherwise.} \end{cases} \tag{S28}$$

Using these expressions, we can compute the average squared epistatic coefficients restricted to the sets of mutations defined by C as

$$\overline{\epsilon^2_{UC}} = \frac{1}{\alpha^{\ell-|U|}}(E_{UC}f)^T(E_{UC}f) = \frac{1}{\alpha^{\ell-|U|}}f^TE^T_{UC}E_{UC}f = \frac{1}{\alpha^{\ell-|U|}}f^T\Delta^{(UC)}f, \tag{S29}$$

where $\Delta^{(UC)}$ can be easily computed by using the Kronecker factorization of E_{UC}

$$\Delta^{(UC)} = \bigotimes_i^{\ell} \begin{cases} \Delta_i^{c_1,c_2} & \text{if } i \in U \\ I & \text{if } i \notin U. \end{cases} \tag{S30}$$

The factor $\Delta_i^{c_1,c_2} = (E_i^{c_1,c_2})^T E_i^{c_1,c_2}$ takes the form

$$\Delta_i^{c_1,c_2}(x,x') = \begin{cases} 1 & \text{if } (x_i = c_1 \wedge x'_i = c_1) \vee (x_i = c_2 \wedge x'_i = c_2) \\ -1 & \text{if } (x_i = c_1 \wedge x'_i = c_2) \vee (x_i = c_2 \wedge x'_i = c_1) \\ 0 & \text{otherwise.} \end{cases} \tag{S31}$$

It is easy to see that $\sum_{c_1,c_2}\Delta_i^{c_1,c_2} = \alpha P_{\text{add}}$ and that $\Delta^{(U)}$ can be expressed as a sum of $\Delta^{(UC)}$ for all possible combinations of pairs of alleles C at sites U.

$$\sum_C \Delta^{(UC)} = \sum_C \bigotimes_i^{\ell} \begin{cases} \Delta_i^{c_{i1},c_{i2}} & \text{if } i \in U \\ I & \text{if } i \notin U \end{cases} = \bigotimes_i^{\ell} \begin{cases} \sum_C \Delta_i^{c_{i1},c_{i2}} & \text{if } i \in U \\ I & \text{if } i \notin U \end{cases} = \Delta^{(U)}. \tag{S32}$$

Next, we use the E_{UC} to compute the average epistatic coefficients $\overline{\epsilon_{UC}}$ between the set of mutations defined by C at sites U across all possible genetic backgrounds:

$$\overline{\epsilon_{UC}} = \frac{1}{\alpha^{\ell-|U|}}1^TE_{UC}f. \tag{S33}$$

This quantity corresponds to the previously proposed background-averaged epistatic coefficient for parametrizing and describing sequence-function relationships (Faure *et al.* 2024a; Petti *et al.* 2025). As the sign of $\overline{\epsilon_{UC}}$ depends on the ordering of alleles c_{i1} and c_{i2} for each position i, we can use its squared value $\overline{\epsilon_{UC}}^2$ to describe the magnitude of the average epistatic coefficients independently of the choice of reference allele at each position:

$$\overline{\epsilon_{UC}}^2 = \left(\frac{1}{\alpha^{\ell-|U|}}1^TE_{UC}f\right)^2 = \frac{1}{\alpha^{2\ell-2|U|}}f^TE^T_{UC}11^TE_{UC}f. \tag{S34}$$

bioRxiv preprint doi: https://doi.org/10.64898/2026.06.25.734428; this version posted June 28, 2026. The copyright holder for this preprint (which was not certified by peer review) is the author/funder, who has granted bioRxiv a license to display the preprint in perpetuity. It is made available under a CC-BY 4.0 International license.

Let $A_{UC} = \frac{1}{\alpha^{\ell-|U|}} E_{UC}^T 11^T E_{UC}$, so that $\overline{\epsilon_{UC}}^2$ can be expressed as a function of a quadratic form with this matrix given by $\frac{1}{\alpha^{\ell-|U|}} f^T A_{UC} f$. To derive a simple expression for A_{UC} and understand its relationship with other objects, we use the fact that all of the matrices E_{UC} and 11^T can be expressed as Kronecker products of site specific factors:

$$A_{UC} = \frac{1}{\alpha^{\ell-|U|}} E_{UC}^T 11^T E_{UC} = \frac{1}{\alpha^{\ell-|U|}} \bigotimes_i^{\ell} \begin{cases} (E_i^{c_{i1},c_{i2}})^T E_i^{c_{i1},c_{i2}} & \text{if } i \in U \\ I11^T I & \text{if } i \notin U \end{cases} = \bigotimes_i^{\ell} \begin{cases} \Delta_i^{c_{i1},c_{i2}} & \text{if } i \in U \\ \frac{1}{\alpha} 11^T & \text{if } i \notin U. \end{cases} \tag{S35}$$

It is easy to see that A_{UC} is in the subspace defined by genetic interactions of order $|U|$ between sites U since $P_U A_{UC} = A_{UC}$. In fact, if we sum over all possible combinations of mutations C, we obtain a matrix that is proportional to the projection operator into the U-subspace P_U:

$$\sum_C A_{UC} = \sum_C \bigotimes_i^{\ell} \begin{cases} \Delta_i^{c_{i1},c_{i2}} & \text{if } i \in U \\ \frac{1}{\alpha} 11^T & \text{if } i \notin U. \end{cases} = \bigotimes_i^{\ell} \begin{cases} \sum_{c_{i1},c_{i2}} \Delta_i^{c_{i1},c_{i2}} & \text{if } i \in U \\ \frac{1}{\alpha} 11^T & \text{if } i \notin U. \end{cases} = \bigotimes_i^{\ell} \begin{cases} \alpha P_{\text{add}} & \text{if } i \in U \\ P_{\text{con}} & \text{if } i \notin U. \end{cases} = \alpha^{|U|} P_U. \tag{S36}$$

Using this equivalence, we can show that the average squared mean epistatic coefficient for mutations at sites U is proportional to the variance explained by genetic interactions of order $|U|$ between those sites:

$$\begin{aligned} \frac{1}{\binom{\alpha}{2}^{|U|}} \sum_C (\overline{\epsilon_{UC}})^2 &= \frac{1}{\binom{\alpha}{2}^{|U|}} \sum_C \frac{1}{\alpha^{\ell-|U|}} f^T A_{UC} f = \frac{1}{\binom{\alpha}{2}^{|U|} \alpha^{\ell-|U|}} f^T \left(\sum_C A_{UC} \right) f = \frac{1}{\binom{\alpha}{2}^{|U|} \alpha^{\ell-|U|}} f^T \left(\sum_C A_{UC} \right) f \\ &= \frac{1}{\binom{\alpha}{2}^{|U|} \alpha^{\ell-|U|}} \alpha^{|U|} f^T P_U f = \frac{2^{|U|}}{\alpha^{\ell}} \left(\frac{\alpha}{\alpha-1} \right)^{|U|} \text{Var}[f_U] \end{aligned} \tag{S37}$$

Finally, we can derive the variance over local epistatic coefficients between mutations at sites U by using the relationship between the variance and the raw second moments $\text{Var}[\epsilon_{UC}] = \overline{\epsilon_{UC}^2} - \overline{\epsilon_{UC}}^2$ as well as the average variance across all possible sets of mutations C at sites U and show that it is proportional to the variance explained by genetic interactions of order higher than $|U|$ involving sites U:

$$\begin{aligned} \text{Var}[\epsilon_U] &= \frac{1}{\binom{\alpha}{2}^{|U|}} \sum_C \text{Var}[\epsilon_{UC}] = \frac{1}{\binom{\alpha}{2}^{|U|}} \sum_C \left(\overline{\epsilon_{UC}^2} - \overline{\epsilon_{UC}}^2 \right) = \frac{1}{\binom{\alpha}{2}^{|U|}} \sum_C \overline{\epsilon_{UC}^2} - \frac{1}{\binom{\alpha}{2}^{|U|}} \sum_C \overline{\epsilon_{UC}}^2 \\ &= \frac{2^{|U|}}{\alpha^{\ell}} \left(\frac{\alpha}{\alpha-1} \right)^{|U|} \left(\text{Var}^{(\text{U})}[\text{f}] - \text{Var}[f_U] \right) = \frac{2^{|U|}}{\alpha^{\ell}} \left(\frac{\alpha}{\alpha-1} \right)^{|U|} \text{Var}^{(\text{U})}_{\text{k}>|\text{U}|}[\text{f}] \, . \end{aligned} \tag{S38}$$

Covariance function between sequences differing at subsets of sites

In addition to characterizing interaction structure through squared epistatic coefficients or epistatic variances of specific subsets of sites U, it is often useful to summarize an *arbitrary* landscape f by how similar fitness values are for sequences that differ at particular sets of sites. In particular, let $H(x, x')$ be the subset of sites at which two sequences x and x' differ

$$H(x, x') = \{ i \in S : x_i \neq x'_i \}. \tag{S39}$$

We define the covariance function for a mismatch set D as

$$C_f(D) = \frac{1}{N_D} \sum_{(x,x'):H(x,x')=D} \left(f(x) - \bar{f} \right) \left(f(x') - \bar{f} \right), \tag{S40}$$

where $\bar{f} = \alpha^{-\ell} \sum_x f(x)$ and $N_D = \alpha^{\ell} (\alpha - 1)^{|D|}$ is the number of sequence pairs that differ at sites D.

$C_f(D)$ can be expressed as a quadratic form $C_f(D) = \frac{1}{N_D} (f - \bar{f})^T A_D (f - \bar{f})$, where A_D is an $\alpha^{\ell} \times \alpha^{\ell}$ matrix given by

$$A_D(x, x') = \begin{cases} 1 & \text{if } H(x, x') = D \\ 0 & \text{if } H(x, x') \neq D. \end{cases} \tag{S41}$$

This matrix can be written as a Kronecker product of single-site matrices, enabling efficient computation of this quantity without explicit evaluation of the matrices A_D (Martí-Gómez *et al.* 2026b):

$$A_D = \bigotimes_{i=1}^{\ell} \begin{cases} 11^T - I & \text{if } i \in D \\ I & \text{if } i \notin D. \end{cases} \tag{S42}$$

Moreover, if we consider the decomposition of f into its orthogonal components $f_U = P_U f$ such that $f = \sum_{U \in \mathcal{P}(S)} f_U$, we can express the covariance function as a sum of covariance over all the possible U-components:

$$C_f(D) = \frac{1}{N_D} \left(\sum_{U \in \mathcal{P}(S)} P_U f \right)^T A_D \left(\sum_{U \in \mathcal{P}(S)} P_U f \right) = \frac{1}{N_D} \sum_{U \in \mathcal{P}(S)} f_U^T P_U A_D P_U f_U = \sum_{U \in \mathcal{P}(S)} C_{f_U}(D). \tag{S43}$$

bioRxiv preprint doi: https://doi.org/10.64898/2026.06.25.734428; this version posted June 28, 2026. The copyright holder for this preprint (which was not certified by peer review) is the author/funder, who has granted bioRxiv a license to display the preprint in perpetuity. It is made available under a CC-BY 4.0 International license.

Next, we note that $P_U A_D P_U$ can be easily calculated through multiplication of their Kronecker factors individually:

$$P_U^T A_D P_U = \bigotimes_{i=1}^{\ell} \begin{cases} -P_{\text{add}} & \text{if } i \in D \wedge i \in U \\ (\alpha-1)P_{\text{con}} & \text{if } i \in D \wedge i \notin U \\ P_{\text{add}} & \text{if } i \notin D \wedge i \in U \\ P_{\text{con}} & \text{if } i \notin D \wedge i \notin U. \end{cases} \tag{S44}$$

In fact, this can be summarized as $P_U^T A_D P_U = (-1)^{|D\cap U|}(\alpha-1)^{|D|-|D\cap U|} P_U$. Since f_U is in the U-subspace, $P_U f_U = f_U$ and thus

$$\begin{aligned} C_{f_U}(D) &= \frac{1}{N_D} f_U^T P_U A_D P_U f_U = \left(\frac{1}{\alpha^\ell (\alpha-1)^{|D|}}\right)\left(f_U^T (-1)^{|D\cap U|}(\alpha-1)^{|D|-|D\cap U|} P_U f_U\right) \\ &= \alpha^{-\ell}(-1)^{|D\cap U|}(\alpha-1)^{-|D\cap U|} f_U^T f_U = \frac{||f_U||^2}{(\alpha-1)^{|U|}} \alpha^{-\ell}(-1)^{|D\cap U|}(\alpha-1)^{|U|-|D\cap U|} \\ &= \frac{||f_U||^2}{(\alpha-1)^{|U|}} w_U(D). \end{aligned} \tag{S45}$$

Here, we denote by $w_U(D)$ the contribution of genetic interactions among sites U to the covariance between sequences that differ at sites D. We refer to these quantities as subset-resolved covariance weights and they are related to the classical Krawtchouk polynomials $w_k(d)$ arising in the Fourier analysis of fitness landscapes (Stadler 1996; Zhou *et al.* 2022). In particular, the functions $w_k(d)$, which depend only on the Hamming distance $d = |D|$, are recovered by summing $w_U(D)$ over all subsets U of size k,

$$w_k(d) = \sum_{U:|U|=k} w_U(D), \qquad \text{for any } D \text{ such that } |D| = d.$$

Gaussian random field landscapes

In the previous sections, we have explained different ways to characterize the patterns of epistatic interactions in a given fitness landscape f. Here we aim to define a probabilistic ensemble of fitness landscapes via a Gaussian distribution $\mathrm{p}(f)$. This Gaussian distribution can be used as a theoretical random fitness landscape model, similar to the classical random fitness landscape models e.g. Rough Mount Fuji, NK or House of Cards landscapes (Kingman 1978; Kauffman and Weinberger 1989; Aita and Husimi 1998), or as a prior distribution for Gaussian process inference

$$\mathrm{p}(f) = \mathcal{N}(\mu, K), \tag{S46}$$

as previously proposed (Zhou *et al.* 2022, 2025). Here, we generally assume that the prior Gaussian distribution has zero mean i.e. $\mu = 0$. To define this prior, let Q_U be an $\alpha^\ell \times (\alpha-1)^{|U|}$-dimensional matrix containing an orthonormal basis for the subspace defined by $|U|$-way interactions among the sites U (i.e. the column space of P_U). We assume that the coefficients for these basis vectors are drawn independently from a zero-mean Gaussian with variance λ_U. In particular, let b_U be the $(\alpha-1)^{|U|}$-dimensional vector of coefficients:

$$b_U \sim \mathcal{N}(0, \lambda_U I). \tag{S47}$$

If we then define $f = \sum_{U\in\mathcal{P}(S)} Q_U b_U$, it is easy to see that f is also a zero-mean Gaussian distribution with covariance matrix K given by

$$K = \mathbb{E}_f[ff^T] = \mathbb{E}_b\left[\left(\sum_{U\in\mathcal{P}(S)} Q_U b_U\right)\left(\sum_{U'} Q_{U'} b_{U'}\right)^{\mathsf{T}}\right] = \sum_{U,U'} Q_U\, \mathbb{E}_b\left[b_U b_{U'}^{\mathsf{T}}\right] Q_{U'}^{\mathsf{T}} = \sum_{U\in\mathcal{P}(S)} \lambda_U Q_U Q_U^{\mathsf{T}} = \sum_{U\in\mathcal{P}(S)} \lambda_U P_U. \tag{S48}$$

Since the columns of Q_U are orthonormal, P_U corresponds to the projection matrix into the function subspace spanned by interactions between sites in U, as given by Eq. S4. Here, we note that the entries of $P_U(x, x')$ only depend on the Hamming distance between sequences x, x' at sites in U, but not on the alleles at which they match or differ. Thus, the covariance matrix $K(x, x')$ only depends on the subset of sites at which x and x' differ.

Moreover, the variance of the regression coefficients λ_U for each subset of sites U determines the expected variance explained by interactions between exactly sites in U and is given by $\mathbb{E}_{f\sim\mathcal{N}(0,K)}\left[||f_U||^2\right] = (\alpha-1)^{|U|}\lambda_U$.

$$\mathbb{E}_f\left[||f_U||^2\right] = \mathbb{E}_f\left[f_U^T f_U\right] = \mathbb{E}_f\left[(P_U f)^T (P_U f)\right] = \mathbb{E}_f\left[f^T P_U f\right]. \tag{S49}$$

We note that samples from the random field model $f \sim \mathcal{N}(0, K)$ can be drawn by first drawing $z \sim \mathcal{N}(0, I)$ and then computing $f = K^{\frac{1}{2}} z$, such that

$$\mathbb{E}_f\left[f^T P_U f\right] = \mathbb{E}_z\left[(K^{\frac{1}{2}} z)^T P_U (K^{\frac{1}{2}} z)\right]. \tag{S50}$$

Moreover, as the columns of P_U are eigenvectors of K with eigenvalue λ_U and P_U matrices are orthogonal to each other, $K^{\frac{1}{2}} = \sum_{U\in\mathcal{P}(S)} \sqrt{\lambda_U} P_U$ and thus

$$\mathbb{E}_f\left[||f_U||^2\right] = \mathbb{E}_z\left[\lambda_U z^T P_U z\right] = \lambda_U \operatorname{tr}(P_U) = \lambda_U \sum_x P_U(x,x) = \lambda_U \alpha^\ell \left(\alpha^{-\ell}(\alpha-1)^{|U|}\right) = (\alpha-1)^{|U|}\lambda_U. \tag{S51}$$

bioRxiv preprint doi: https://doi.org/10.64898/2026.06.25.734428; this version posted June 28, 2026. The copyright holder for this preprint (which was not certified by peer review) is the author/funder, who has granted bioRxiv a license to display the preprint in perpetuity. It is made available under a CC-BY 4.0 International license.

A prior distribution for fitness landscapes

Because for sequences of length ℓ these priors are defined by there 2^ℓ values λ_U, defining and interpreting this large number of the parameters can be challenging. Here, we use the relationship between the $\Delta^{(U)}$ and the projection operators into the U-subspace shown in Eq. S21 to define a simplified prior where the λ_U corresponding to interactions of order higher than P are parametrized via the different $\binom{\ell}{P}$ values of a_U:

$$\lambda_U = \begin{cases} \tilde{\lambda}_U & \text{if } |U| < P \\ \frac{1}{\alpha^{|U|} \sum_{U' \subseteq U} a_{U'}} & \text{if } |U| >= P. \end{cases} \tag{S52}$$

To understand the role of the parameters a_U in the prior, we can write it as

$$\mathrm{p}(f) \propto e^{-f^T K^{-1} f} = e^{-\sum_{U:|U|<P} \frac{1}{\tilde{\lambda}_U} f^T P_U f - \sum_{U:|U|=P} \frac{a_U}{s_U} f^T \Delta^{(U)} f}, \tag{S53}$$

where s_U is the number of local epistatic coefficients between sites in U and the value of a_U modulates how much the prior penalizes these coefficients for a given f. Interestingly, one can see that as $a_U \to \infty$, λ_U approaches 0 for all function subspaces explained by interactions involving the sites in U, essentially removing interactions of order equal or higher than $|U|$ involving the set of sites U. For instance, for $P = 2$, setting $a_{ij} = \infty$ enforces the assumption that there are no genetic interactions between sites i and j or, equivalently, that the effects of mutations at site i never change when introducing an additional mutation at site j. This property allows us to define prior distributions where genetic interactions of order higher than $|U|$ are allowed, but are constrained to specific sets of sites. Similarly, if we set all the $\tilde{\lambda}_U = c$ for $|U| < P$ we see that the first sum in the exponent of Equation S53 becomes proportional to the squared norm of the projection of f into the null space of $\Delta^{(P)}$. Taking the limit $c \to \infty$ is then equivalent to imposing a uniform prior on these directions, and for the special case $P = 2$ we recover Equation 4 in the main text.

Next, we consider the expected average squared epistatic coefficients involving sites U under the prior distribution $f \sim \mathcal{N}(0, K)$, parameterized by the lower-order variances $\tilde{\lambda}_U$ and a_U for $|U| \geq P$. To draw samples from this prior, we can first sample $z \sim \mathcal{N}(0, I)$ and then set $f = K^{1/2} z$, so that $\mathrm{Cov}[f] = K^{1/2}(K^{1/2})^\intercal = K$ (for any matrix square root $K^{1/2}$). One way to define the matrix square root is via its known eigendecomposition $K^{1/2} = \sum_{U \in \mathcal{P}(S)} \sqrt{\lambda_U} P_U$. Then, we consider the average squared epistatic coefficient for any f and compute its expectation when f are drawn from the prior distribution as follows

$$\mathbb{E}_f \left[\frac{1}{s_U} f^T \Delta^{(U)} f \right] = \mathbb{E}_z \left[\frac{1}{s_U} z^T (K^{1/2})^\intercal \Delta^{(U)} K^{1/2} z \right] = \frac{1}{s_U} \mathrm{tr}\Big((K^{1/2})^\intercal \Delta^{(U)} K^{1/2} \Big). \tag{S54}$$

We compute the $K^{1/2} \Delta^{(U)} K^{1/2}$ product by using the known eigendecompositions of K and $\Delta^{(U)}$:

$$\begin{aligned} \mathbb{E}_f \left[\frac{1}{s_U} f^T \Delta^{(U)} f \right] &= \frac{1}{s_U} \mathrm{tr} \left(\sum_{U':U \subseteq U'} \lambda_{U'} \alpha^U P_U \right) = \frac{1}{s_U} \sum_{U':U \subseteq U'} \lambda_{U'} \alpha^{|U|} \mathrm{tr}(P_{U'}) \\ &= \frac{1}{s_U} \sum_{U':U \subseteq U'} \frac{1}{\alpha^{|U'|} \sum_{U'' \subseteq U'} a_{U''}} \alpha^{|U|} (\alpha - 1)^{|U'|} = \frac{\alpha^{|U|}}{s_U} \sum_{U':U \subseteq U'} \left(\frac{\alpha - 1}{\alpha} \right)^{|U'|} \frac{1}{\sum_{U'' \subseteq U'} a_{U''}} \\ &= \frac{\alpha^{|U|} 2^{|U|}}{\alpha^\ell (\alpha - 1)^{|U|}} \sum_{U':U \subseteq U'} \left(\frac{\alpha - 1}{\alpha} \right)^{|U'|} \frac{1}{\sum_{U'' \subseteq U'} a_{U''}} \\ &= \left(\frac{\alpha^\ell}{2^{|U|}} \left(\frac{\alpha - 1}{\alpha} \right)^{|U|} \right)^{-1} \sum_{U':U \subseteq U'} \left(\frac{\alpha - 1}{\alpha} \right)^{|U'|} \frac{1}{\sum_{U'' \subseteq U'} a_{U''}}. \end{aligned} \tag{S55}$$

Thus, we can see that the expected average squared local epistatic coefficient for sites U does not depend only on the value a_U, but also on all other $a_{U'}$ such that both sets have at least one site in common ($|U \cap U'| > 0$). For instance, if we let $a_U \to \infty$ for a particular U, $\lambda_{U'}$ for all U' that include the whole set of sites in U will be set to zero, decreasing the expected average squared epistatic coefficients for sites involving sites in U'.

Relationship with the Connectedness Model

In this section, we review the Connectedness Model and its relationship to Local Epistasis Regression. The Connectedness Model was first introduced as a Gaussian random field model by Reddy and Desai (2021) to allow different loci to have different probability of being involved in epistatic interactions with mutations at other sites. Then, it was used as a prior distribution in a Gaussian process model, uncovering the set of sites that are more strongly involved in epistatic interactions and using that information for inference of high-dimensional empirical fitness landscapes (Zhou *et al.* 2025). Here, we show that the Connectedness Model can be derived as a particular case of Eq. S48 as a function of the variance associated to the constant component $\tilde{\lambda}_0$ and the variance associated to the additive contribution of each site $\tilde{\lambda}_i$

$$\lambda_U = \prod_{i \in U} \tilde{\lambda}_i \prod_{i \notin U} \tilde{\lambda}_0. \tag{S56}$$

bioRxiv preprint doi: https://doi.org/10.64898/2026.06.25.734428; this version posted June 28, 2026. The copyright holder for this preprint (which was not certified by peer review) is the author/funder, who has granted bioRxiv a license to display the preprint in perpetuity. It is made available under a CC-BY 4.0 International license.

If we plug this into Eq. S48, we can see that the resulting kernel can be expressed as a product of site-specific kernels

$$K = \sum_{U \in \mathcal{P}(S)} \lambda_U P_U = \sum_{U \in \mathcal{P}(S)} \prod_{i \in U} \tilde{\lambda}_i \prod_{i \notin U} \tilde{\lambda}_0 \bigotimes_{i=1}^{\ell} P_{i \in U} = \sum_{U \in \mathcal{P}(S)} \bigotimes_{i=1}^{\ell} \begin{cases} \tilde{\lambda}_0 P_{\text{con}} & i \notin U \\ \tilde{\lambda}_i P_{\text{add}} & i \in U \end{cases}$$
$$= \bigotimes_{i=1}^{\ell} (\tilde{\lambda}_0 P_{\text{con}} + \tilde{\lambda}_i P_{\text{add}}) = \bigotimes_{i=1}^{\ell} K_i, \tag{S57}$$

with entry-wise formula given by

$$k(x, x') = \alpha^{-\ell} \prod_{i: x_i = x_i'} (\tilde{\lambda}_0 + \tilde{\lambda}_i(\alpha - 1)) \prod_{i: x_i \neq x_i'} (\tilde{\lambda}_0 - \tilde{\lambda}_i). \tag{S58}$$

This kernel can be reparametrized as a function of the prior variance σ^2 and the correlation under this kernel parametrized by $\mu_i = \tilde{\lambda}_i / \tilde{\lambda}_0$ assuming $\tilde{\lambda}_0 > 0$ as in Zhou *et al.* (2025):

$$k(x, x') = \sigma^2 \prod_{i: x_i \neq x_i'} \frac{1 - \mu_i}{1 + \mu_i(\alpha - 1)}. \tag{S59}$$

This construction shows that the variance explained by epistatic interactions under the Connectedness Model is completely specified by the variance explained by the additive contribution of each individual site. In contrast, Local Epistasis Regression allows arbitrary relationships between the additive and pairwise contribution of individual sites and pairs of sites, but the variance for higher-order interactions is fully specified by the the variance associated to pairwise interactions between specific pairs of sites (Eq. S52). These models also make different assumptions on how the variances associated to lower-order interactions combine to specify the variances associated to higher-order interactions. In particular, in the Connectedness Model these variances combine multiplicatively (Eq. S56), whereas in Local Epistasis Regression they are proportional to the harmonic mean of the variances associated to pairwise interactions between each possible pair of sites in U

$$\lambda_U = \frac{1}{\sum_{i<j \in U} \frac{1}{\tilde{\lambda}_{ij}}}, \tag{S60}$$

where $\tilde{\lambda}_{ij} = \frac{1}{\alpha^2 a_{ij}}$.

Inference of fitness landscapes under the prior

In this section, we review how to do Gaussian process inference of a complete fitness landscape f from high throughput experimental data (Martí-Gómez *et al.* 2026b). We start by defining a Gaussian prior distribution over the space of possible fitness landscapes $p(f) \sim \mathcal{N}(0, K)$ that assigns higher probability to fitness landscapes that we believe are more plausible *a priori*.

Let y be an n-dimensional vector of measurements with known experimental Gaussian error given by the variance n-dimensional vector y_{var} for a subset of $n \leq \alpha^\ell$ sequences X. As both the prior distribution and the likelihood function are Gaussian, the posterior distribution is also multivariate Gaussian with closed form analytical solution (Rasmussen and Williams 2008) for the mean

$$\hat{f} = K_{*X}(K_{XX} + D_{var})^{-1} y \tag{S61}$$

and covariance matrix

$$K - K_{*X}(K_{XX} + D_{var})^{-1} K_{X*}, \tag{S62}$$

where K_{XX}, K_{*X}, K_{X*} are submatrices of K indexed by sequences X and $*$, where $*$ represents all possible sequences, and D_{var} is a diagonal matrix with the known experimental variances y_{var} along the diagonal.

Despite the simplicity of the solution, practical evaluation of these expressions becomes challenging as the number of observations increases. Traditional approaches rely to computation of the Cholesky decomposition of the $K_{XX} + D_{var}$ matrix to then use efficient triangular solves to compute the solutions to the linear systems rather than using direct matrix inversion for higher numerical stability. However, the algorithm for computing this decomposition is $O(n^3)$ and cannot be parallelized, which has traditionally limited the applicability of Gaussian process models to datasets with at most few thousand data points (Rasmussen and Williams 2008). In previous work, we have circumvented this limitation by leveraging the mathematical properties of the specific precision or kernel matrices, which could be expressed as polynomials in the Laplacian of the Hamming graph representing sequence space (Zhou and McCandlish 2020; Zhou *et al.* 2022; Chen *et al.* 2021; Martí-Gómez *et al.* 2026b). This property allowed us to encode these matrices as linear operators that allow computing matrix-vector products efficiently without explicitly storing them in memory, and use these operators together with iterative methods for solving systems of linear equations to scale these methods up to a few million data points.

Here we use a similar strategy by finding a representation of the kernel matrices that enables efficient computation of matrix-vector products without explicitly constructing them in memory, even if the kernel matrices presented here cannot be represented as polynomials in the Laplacian of the Hamming graph.

In the case of the Connectedness Model (Zhou *et al.* 2025), the kernel can in fact be expressed as a Kronecker product of ℓ site-specific $\alpha \times \alpha$ matrices (Eq. S57), which enables fast computation of matrix-vector products by leveraging the mixed-product property, which reduces the complexity to that of computing ℓ products of an $\alpha \times \alpha$ matrix with an $\alpha \times \alpha^{\ell-1}$ matrix (Martí-Gómez *et al.* 2026b,a).

bioRxiv preprint doi: https://doi.org/10.64898/2026.06.25.734428; this version posted June 28, 2026. The copyright holder for this preprint (which was not certified by peer review) is the author/funder, who has granted bioRxiv a license to display the preprint in perpetuity. It is made available under a CC-BY 4.0 International license.

In the case of Local Epistasis Regression, the kernel matrix can be expressed as the sum of 2^ℓ matrices, each of which is Kronecker factorizable

$$K = \sum_{U \in \mathcal{P}(S)} \lambda_U \bigotimes_{i=1}^{\ell} P_{i\in U}. \tag{S63}$$

While this enables computation of matrix-vector products with a total of $2^\ell \ell$ $\alpha \times \alpha$ by $\alpha \times \alpha^{\ell-1}$ matrix products, the computational burden of these calculations is much larger compared with previous approaches e.g. requires 2^ℓ times the computation needed in the Connecntedness model. However, here we note that all of the 2^ℓ kernel matrices represent different combinations of only two different Kronecker factors P_{con} and P_{add}, which allow us to re-use parts of the computation. In particular, we can decompose any P_U as follows

$$P_U = \bigotimes_{i=1}^{\ell} P_{i\in U} = (P_{1\in U} \otimes I \otimes \cdots \otimes I)(I \otimes P_{2\in U} \otimes \cdots \otimes I)\ldots(I \otimes I \otimes \cdots \otimes P_{\ell \in U}) = M_{1\in U} M_{2\in U} \ldots M_{\ell\in U}. \tag{S64}$$

While the M matrices are still Kronecker products of ℓ matrices, $\ell - 1$ of the factors correspond to the identity matrix and thus leave the matrices they act on unchanged, reducing the computation to a single $\alpha \times \alpha$ by $\alpha \times \alpha^{\ell-1}$ matrix products. Importantly, P_U matrices differing only at the factor at the first site can be computed with a single additional operation from the same intermediate result

$$P_U v = M_{1\in U}(M_{2\in U} \ldots M_{\ell \in U} v). \tag{S65}$$

The intermediate results can also be computed in the same fashion

$$M_{2\in U} M_{3\in U} \ldots M_{\ell\in U} v = M_{2\in U}(M_{3\in U} \ldots M_{\ell\in U} v) \tag{S66}$$

so that the computation can be shared with products of the same vector with other $P_{U'}$ for a different subset of sites U'. These computational dependencies can be represented by a bifurcating tree where nodes represent α^ℓ-dimensional vectors and edges represent matrix-vector products with specific $M_{i\in U}$ matrices. The vector v is located at the root of the tree, allowing computation of the intermediate vectors at each of the nodes of the tree up to the tips containing all the $P_U v$ for every possible U. This algorithm reduces the total number of operations from $2^\ell \ell$ to $2^\ell \sum_{i=0}^{\ell} 2^{-i}$. This series converges relatively fast to $2^{\ell+1}$, resulting in an approximate $\ell/2$-fold speedup even when ℓ is small. For instance, for $\ell = 8$, the number of matrix-matrix products goes from $8 \times 2^8 = 2048$ under the naive approach to 510, nearly achieving the expected 4-fold increase in computational efficiency.

Hyperparameter optimization

In order to infer a fitness landscape under a given prior distribution, we must first choose the parameters that define the properties of the prior, also known as hyperparameters. Here, we generally consider prior distributions defined over the space of possible fitness landscapes f defined by a kernel function $k(x, x')$ that returns the covariance for any pair of sequences x and x' given by

$$k(x, x') = \sum_{U \in \mathcal{P}(S)} \lambda_U P_U(x, x'), \tag{S67}$$

where $P_U(x, x')$ is the covariance due to interactions between exactly U sites between sequences x and x', which depends only on the combination of sites at which they differ $P_U(x, x') = w_U(H(x, x'))$; and where the parameters λ_U can be free or a function of a smaller set of parameters generally called θ ($\lambda_U = g(\theta)$) e.g. Eq. S52 in Local Epistasis Regression and Eq. S56 in the Connectedness Model.

In this work, we use a strategy known as kernel alignment (Wang *et al.* 2015; Zhou *et al.* 2022) or Haseman-Elston regression (Haseman and Elston 1972), in which the prior covariance approximates as closely as possible the patterns of covariance in the empirical data. Specifically, this is done by finding the parameter values $\hat{\theta}$ that minimize the Frobenius norm of the difference between the prior predictive covariance $K_{XX} + D_{var}$ and the empirical second moment matrix yy^T:

$$\hat{\theta} = \arg\min_{\theta} \left\| yy^T - (K_{XX}(\theta) + D_{var}) \right\|_F^2. \tag{S68}$$

Naively solving this minimization problem is challenging, as we need to work with $n \times n$ matrices, where the number of measured sequences n can be in the order of hundreds of thousands to millions. However, as the prior covariance between two sequences only depends on the set of sites at which they differ, the dimensionality of the problem can be reduced to a more manageable 2^ℓ-dimensional weighted least squares problem as follows:

$$\begin{aligned}
\left\| yy^T - (K_{XX}(\theta) + D_{var}) \right\|_F^2 &= \sum_{x \in X} \left[(y_x^2 - y_{x,var}) - k_\theta(\varnothing) \right]^2 + \sum_{D \neq set} \sum_{x,x': H(x,x') = D} [y_x y_{x'} - k_\theta(D)]^2 \\
= N_\varnothing \left[t(\varnothing) - k_\theta(\varnothing) \right]^2 + \sum_{x\in X} \left[(y_x^2 - y_{x,var}) - t(\varnothing) \right]^2 &+ \sum_{D \neq \varnothing} N_D \left[t(D) - k_\theta(D) \right]^2 + \sum_{D \neq \varnothing} \sum_{x,x': H(x,x') = D} [y_x y_{x'} - t(D)]^2 \\
&= \sum_{D} N_D \left[t(D) - k_\theta(D) \right]^2 + \sum_{x \in X} \left[(y_x^2 - y_{x,var}) - t(\varnothing) \right]^2 + \sum_{D \neq \varnothing} \sum_{x,x': H(x,x') = D} [y_x y_{x'} - t(D)]^2.
\end{aligned} \tag{S69}$$

bioRxiv preprint doi: https://doi.org/10.64898/2026.06.25.734428; this version posted June 28, 2026. The copyright holder for this preprint (which was not certified by peer review) is the author/funder, who has granted bioRxiv a license to display the preprint in perpetuity. It is made available under a CC-BY 4.0 International license.

Since the last two terms are independent of θ, we can find by optimal hyperparameter values $\widehat{\theta}$ simply as

$$\widehat{\theta} = \arg\min_{\theta} \sum_{D\in\mathcal{P}(S)} N_D \left[t(D) - \sum_{U\in\mathcal{P}(S)} \lambda_U(\theta) w_U(D) \right]^2, \tag{S70}$$

where $t(D)$ corresponds to the second moment, related with the empirical autocovariance function $c(D)$ ($t(D) = c(D) + \bar{y}^2$) given by

$$c(D) = \begin{cases} \frac{1}{n}\sum_{x\in X}(y_x - \bar{y})^2 - \overline{y_{var}} & D = \emptyset \\ \frac{1}{N_D}\sum_{x,x'\in X:H(x,x')=D}(y_x - \bar{y})(y_{x'} - \bar{y}) & D \neq \emptyset. \end{cases} \tag{S71}$$

bioRxiv preprint doi: https://doi.org/10.64898/2026.06.25.734428; this version posted June 28, 2026. The copyright holder for this preprint (which was not certified by peer review) is the author/funder, who has granted bioRxiv a license to display the preprint in perpetuity. It is made available under a CC-BY 4.0 International license.

Supplementary figures

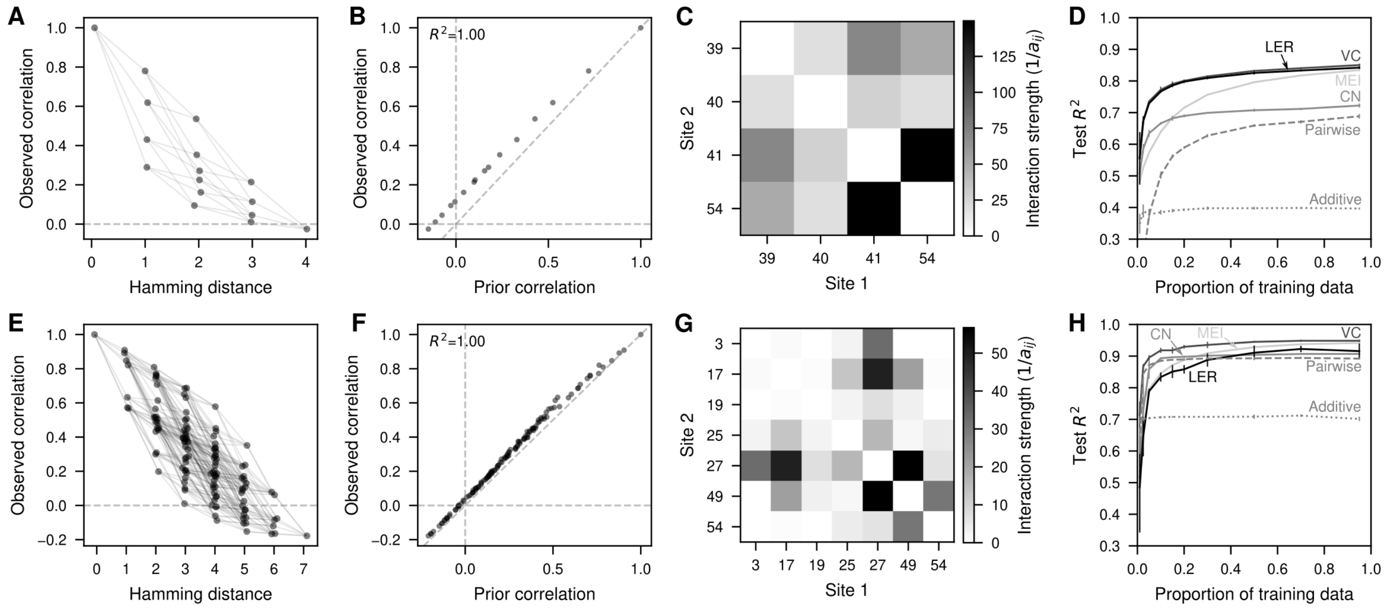

Figure S1 Application of Local Epistasis Regression to protein datasets. (A,E) Correlation in the measured fitness values for pairs of sequences differing at each possible subset of sites D arranged according to the Hamming distance $d = |D|$. Each dot represents a single distance class D and are joined by lines whenever the distance classes differ by a single position from each other. (B,F) Comparison of the observed correlation values in the data and the values under the estimated prior ones using Local Epistasis Regression for every possible distance class D (each dot represents a different D). Correlations were estimated using 80% of the data for training. (C,G) Heatmap representing the inferred model hyperparameters as $1/a_{ij}$ for every pair of sites i, j highlighting the patterns of genetic interactions across sites under the prior. (D,H) Predictive performance evaluated by the R^2 between the predicted and the measured fitness of held-out test sequences when using different amounts of training data for different models (MEI: Minimum Epistasis Interpolation, VC: Variance Component regression, CN: Connectedness Model regression, LER: Local Epistasis Regression). Predicted values are the maximum a posteriori estimate given by each method, which is equal to the posterior mean $\hat{f}$. Error bars represent the standard deviation across 3 different random samples for each fraction of training data. Each row represents a fitness landscape: GB1 (A,B,C,D); FYN-SH3 (E,F,G,H).

bioRxiv preprint doi: https://doi.org/10.64898/2026.06.25.734428; this version posted June 28, 2026. The copyright holder for this preprint (which was not certified by peer review) is the author/funder, who has granted bioRxiv a license to display the preprint in perpetuity. It is made available under a CC-BY 4.0 International license.

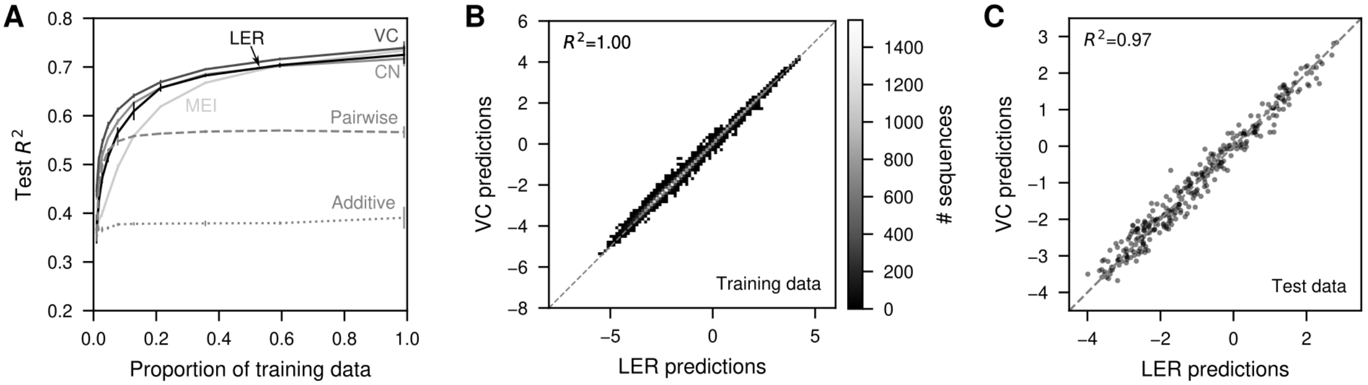

Figure S2 Comparison of model predictions on the self-splicing intron dataset. (A) Predictive performance evaluated by the R^2 between the predicted and the true fitness values of held-out test sequences when using different amounts of training data for different models (MEI: Minimum Epistasis Interpolation, VC: Variance Component regression, CN: Connectedness Model regression, LER: Local Epistasis Regression). Predicted values are the maximum a posteriori estimate given by each method, which is equal to the posterior mean $\widehat{f}$. Error bars represent the standard deviation across 3 different random samples for each fraction of training data. (B) 2D histogram comparing the fitness landscape reconstructions of the self-splicing intron dataset under Local Epistasis Regression (LER) and Variance Component regression (VC). (C) Scatterplot comparing the predictions in held-out sequences of the self-splicing intron dataset under Local Epistasis Regression (LER) and Variance Component Regression (VC).

bioRxiv preprint doi: https://doi.org/10.64898/2026.06.25.734428; this version posted June 28, 2026. The copyright holder for this preprint (which was not certified by peer review) is the author/funder, who has granted bioRxiv a license to display the preprint in perpetuity. It is made available under a CC-BY 4.0 International license.

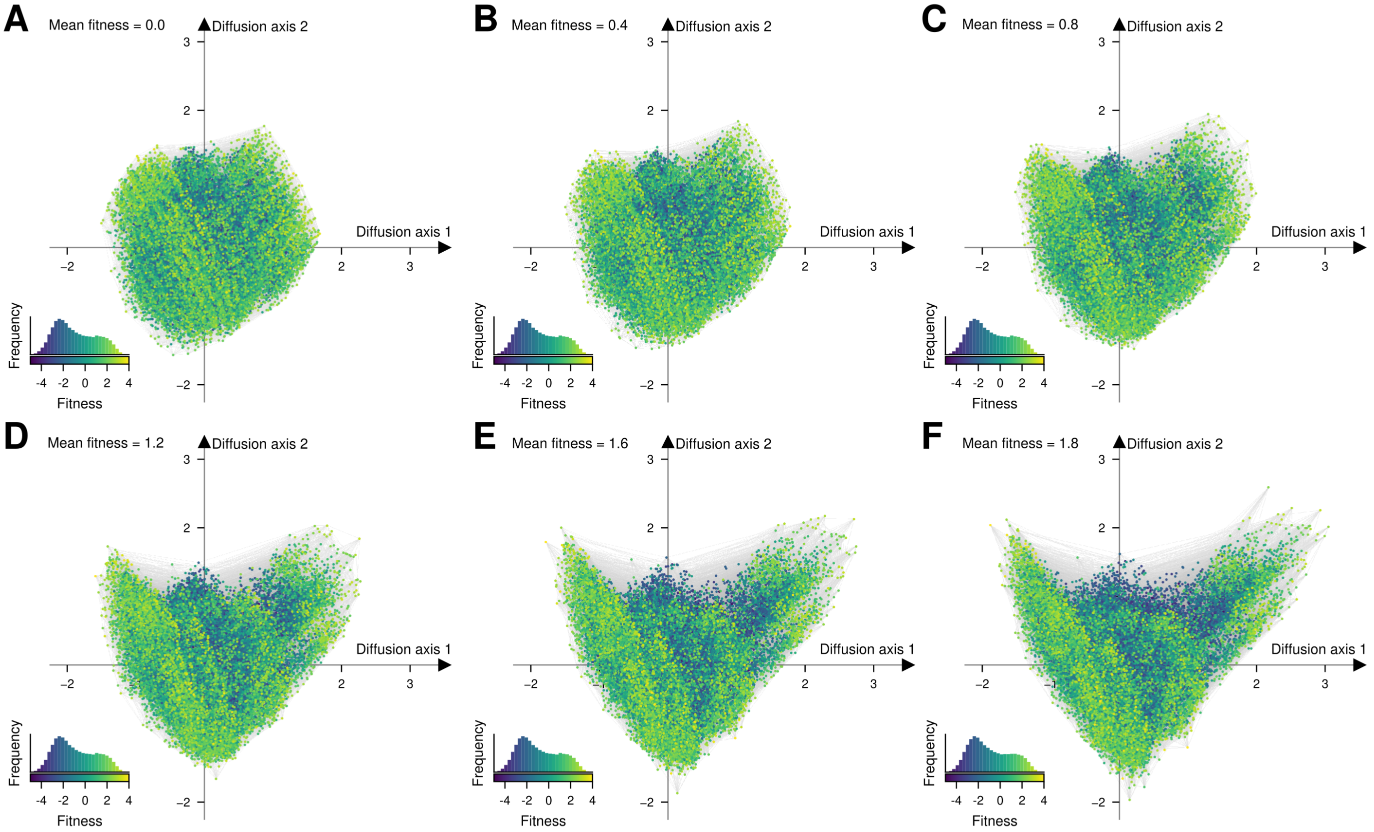

Figure S3 Visualization of the inferred fitness landscape using Local Epistasis Regression under different strengths of selection, where here we quantify the strength of selection by the mean fitness achieved at stationarity, i.e. under long-term purifying selection. Every dot represents one of the possible 4^8 possible sequences and is colored according to the predicted fitness. The inset represents the phenotypic distribution along with their corresponding color in the map. Sequences are laid out according to the first two Diffusion axes and dots are plotted in order according to Diffusion axis 3.

bioRxiv preprint doi: https://doi.org/10.64898/2026.06.25.734428; this version posted June 28, 2026. The copyright holder for this preprint (which was not certified by peer review) is the author/funder, who has granted bioRxiv a license to display the preprint in perpetuity. It is made available under a CC-BY 4.0 International license.

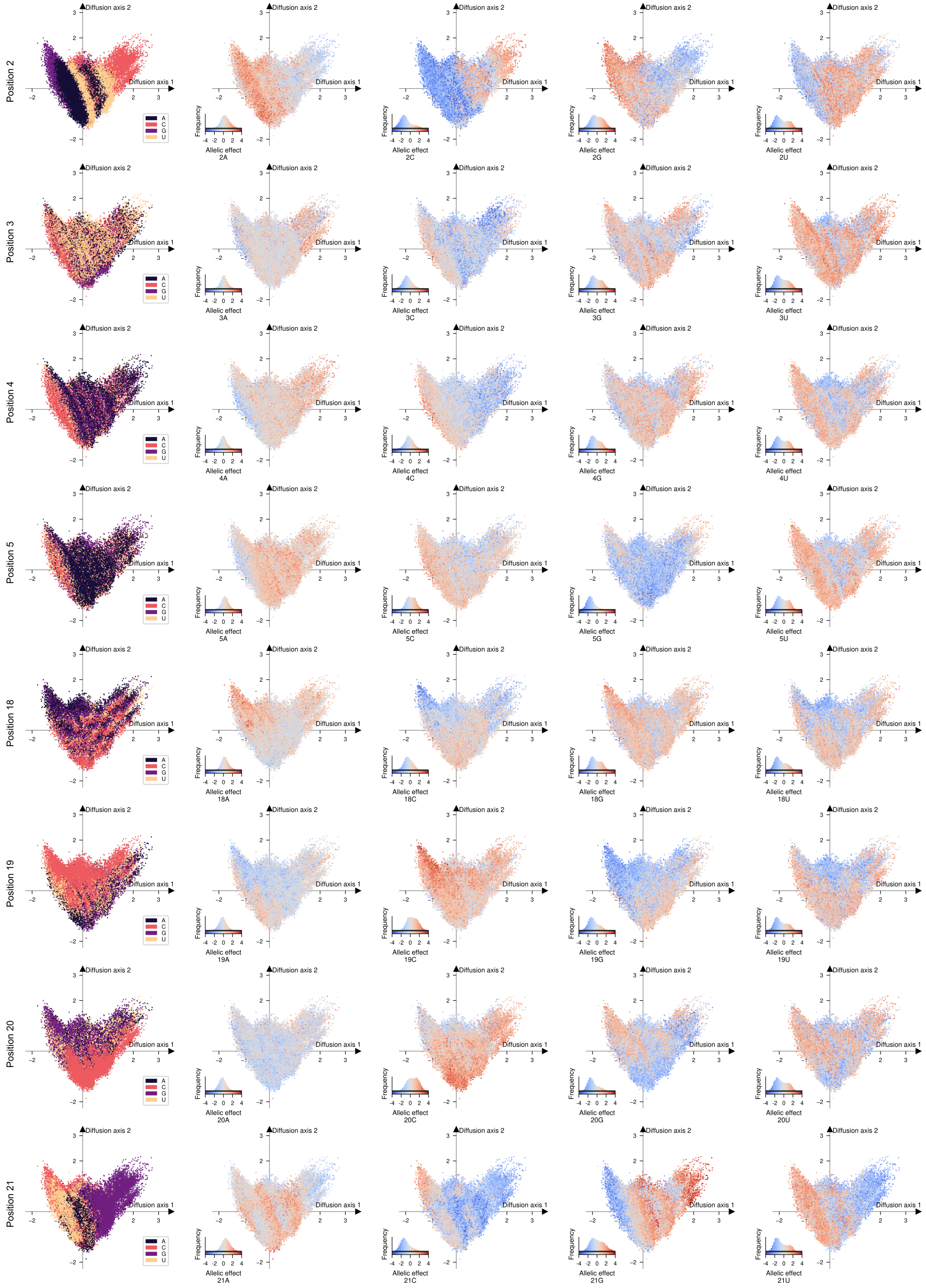

Figure S4 Visualizing alleles and allelic preferences across the fitness landscape visualization. Visualization of the inferred fitness landscape using Local Epistasis Regression. Every dot represents one of the possible 4^8 possible sequences and is colored according to the allele (first column) or the difference in the fitness of the sequence obtained when placing a specific allele at an specifc position relative to the average fitness of the four possible alleles (four last columns). The inset represents the allelic effect distribution along with their corresponding color in the map. Sequences are laid out according to the first two Diffusion axes and dots are plotted in order according to Diffusion axis 3.
